# Relationship between the bony correction angle and mechanical axis change and their differences between closed and open wedge high tibial osteotomy

**DOI:** 10.1186/s12891-020-03703-6

**Published:** 2020-10-12

**Authors:** Takahiro Ogino, Ken Kumagai, Shunsuke Yamada, Tomotaka Akamatsu, Shuntaro Nejima, Masaichi Sotozawa, Yutaka Inaba

**Affiliations:** Department of Orthopaedic Surgery, Yokohama City University Hospital, 3-9 Fukuura, Kanazawa-ku, Yokohama, 236-0004 Japan

**Keywords:** High tibial osteotomy, Opening wedge, Closed wedge, Mechanical axis shift, Correction angle

## Abstract

**Background:**

The purpose of this study was to investigate the relationship between the bony correction angle and mechanical axis change and their differences between closed wedge high tibial osteotomy (CWHTO) and open wedge high tibial osteotomy (OWHTO).

**Methods:**

A total of 100 knees of 89 patients who underwent OWHTO (50 knees) or CWHTO (50 knees) between 2011 and 2015 with a clinical follow-up for 1 year and a radiological follow-up for 1 month were investigated in a case control study. Anteroposterior radiographs of the knee and full-length leg were taken in the standing position using digital acquisition. The femorotibial angle (FTA), % mechanical axis deviation (MAD), % anatomical tibial axis deviation (ATAD), % mechanical tibial axis deviation (MTAD), mechanical medial proximal tibial angle (mMPTA), and joint line convergence angle (JLCA) were measured on preoperative and postoperative radiographs using a dedicated software.

**Results:**

CWHTO resulted in a greater variation between the tibial anatomical and mechanical axes than OWHTO (*P* <  0.05), and a greater soft tissue correction than OWHTO (*P* <  0.05). However, no significant difference was found between CWHTO and OWHTO in the ratio of MAD change to the correction angle. When the osteotomy was planned with the same bony correction angle, %MAD passed more laterally in OWHTO than in CWHTO (*P* <  0.05). These results suggested a lesser valgus bony correction ratio due to greater medial shift of the tibial axis and greater valgus compensation of the soft tissue in CWHTO compared to OWHTO.

**Conclusions:**

The ratio of mechanical axis shift to the correction angle differed in preoperative planning, but postoperative alignment was comparable between opening wedge and closed wedge high tibial osteotomy.

## Background

High tibial osteotomy (HTO) is an established procedure to correct lower limb alignment and to reduce the mechanical force on the affected compartment. Proper overcorrection provides pain relief and subsequent improvement of knee function [1, 2]. Two commonly used procedures for HTO are the lateral closed wedge HTO (CWHTO) and the medial opening wedge HTO (OWHTO). Excellent clinical outcomes have been reported with both techniques, although they each have potential advantages and disadvantages [3–5]. In CWHTO, the advantage is the possibility of large correction, and the disadvantage is the invasive surgical procedure [6]. In OWHTO, the advantage is the less invasive surgical procedure, and the disadvantage is the limited correction angle [7].

Several studies have reported significant differences between CWHTO and OWHTO in radiological variables. A meta-analysis of 28 trials involving 2840 knees showed that OWHTO increased the posterior slope and decreased the patellar height, whereas CWHTO lead to a decreased posterior slope and an unchanged patellar height [8]. A recent prospective randomized study of 70 patients demonstrated that posterior tibial slope and leg length changes were significantly different between CWHTO and OWHTO [9]. However, to date, there have been no reports of a comparison between CWHTO and OWHTO regarding the relationship between the correction angle at the osteotomy site and shift of the mechanical axis. A wedged bone is removed from the lateral cortex in CWHTO, and the proximal tibia is offset laterally. In contrast, the lateral cortex is retained in OWHTO. The amount of lateral shift of the proximal tibia from the anatomical axis differs between CWHTO and OWHTO [10]. That is, the effect of the same bony correction angle on mechanical axis deviation is presumed to differ between CWHTO and OWHTO [11, 12]. This discrepancy between the two HTO procedures needs to be elucidated for accurate surgical planning.

The purpose of this study was to investigate the relationship between the bony correction angle and mechanical axis change and their differences between CWHTO and OWHTO. It was hypothesized that CWHTO shows greater medial shift of the tibial axis and less mechanical axis change than OWHTO with the same bony correction angle.

## Methods

Between January 2012 and September 2013, 84 knees of 74 consecutive patients underwent OWHTO. Between May 2011 and December 2015, 73 knees of 59 consecutive patients underwent CWHTO. Three board-certified orthopaedic surgeons specializing in knee surgery performed both HTO procedures. The inclusion criterion was painful osteoarthritis (OA) localized to the medial compartment of the knee. Exclusion criteria were OA of the lateral compartment, flexion contracture greater than 15°, or a history of inflammatory arthritis, joint infection, or immunosuppressive therapy. The decision for either technique was made preoperatively according to the correction angle based on our institutional protocol [7]. OWHTO was performed in knees with a correction angle of 15° or less, and CWHTO was performed in knees with a correction angle of greater than 15°. Of the 84 knees in the OWHTO group, 34 knees were excluded from the analysis due to being lost to follow-up (8 knees), insufficient data (19 knees), and bone-related complications (lateral hinge fracture, 7 knees). Of the 73 knees in the CWHTO group, 23 knees were excluded from the analysis due to being lost to follow-up (6 knees), insufficient data (14 knees), and bone-related complications (non-union, 3 knees). Therefore, a total of 100 knees of 89 patients (50 knees of 46 patients in the OWHTO group and 50 knees of 43 patients in CWHTO group) were included in this study. Demographic data are shown in Table 1. This retrospective case control study was approved by the institutional review board of Yokohama City University (#B180200061), and written informed consent was obtained from all individual participants included in the study.

**Table 1:** Demographic data

	CWHTO	OWHTO
Number of patients (knees)	43 (50)	46 (50)
Male	15 (17)	14 (16)
Female	28 (33)	32 (34)
Age (years)	63.9 ± 22.9	64.5 ± 21.5
Body mass index (kg/m^2^)	25.4 ± 10.1	24.6 ± 6.2
OA grade^a^ 2/3/4 (knees)	7/25/19	33/11/6

^a^ OA grade modified from Ahlbach’s classification

### Surgical procedure and postoperative management

The amount of angular correction was planned preoperatively aiming to achieve tibiofemoral anatomical valgus of 10° in a one-leg standing radiograph postoperatively.

OWHTO was performed using an anteromedial approach under fluoroscopic guidance. The osteotomy was started 35 mm below the medial articular surface of the tibia. An oblique osteotomy was performed from the medial cortex to the upper third of the proximal tibiofibular joint using biplanar technique, leaving the tibial tuberosity intact. The osteotomized gap was gradually opened and filled with two wedged blocks of β-TCP with 60% porosity (Osferion®, Olympus Terumo Biomaterials. Corp., Tokyo, Japan) and fixed with TomoFix (DePuy Synthes, Zuchwil, Switzerland). In general, healing of the opening gap is required by filling with autologous bone grafting, allogenic bone grafting, syngeneic bone grafting, or no bone grafting, and [13–15]. A β-TCP wedged block with 60% porosity was used as a synthetic bone substitute in the present study, since it shows similar radiographic union and correction maintenance to autologous bone grafting [7, 13], and provides additional initial stability [16], and a large opening gap without bone grafting delays filling of the opening gap [17, 18].

CWHTO was performed using an anterolateral approach under fluoroscopic guidance after fibular osteotomy. The osteotomy was started 30 mm below the lateral articular surface of the tibia. The proximal osteotomy was performed parallel to the tibial plateau, and the distal osteotomy was performed obliquely toward the hinge point of the medial cortex, with a flange to leave the insertion of the patellar tendon with a distal fragment. The osteotomy gap was closed and fixed with an OWL plate (Mizuho Ikakogyo Co., Ltd., Tokyo, Japan).

Patients started a postoperative rehabilitation program [7] including isometric quadriceps and range-of-motion exercises the day after surgery. In CWHTO, a non-weight-bearing regimen was prescribed for 2 weeks, followed by partial weight-bearing exercise, and full weight-bearing exercise was permitted 3 weeks postoperatively. In OWHTO, a non-weight-bearing regimen was prescribed for 1 week, followed by full weight-bearing exercise. Casts or supportive devices were never applied in both procedures.

### Clinical assessment

Clinical evaluation was carried out using the Japanese Orthopaedic Association (JOA) score for knee osteoarthritis [19] before surgery and 1 year after surgery.

### Radiographic assessment

**Fig. 1:**
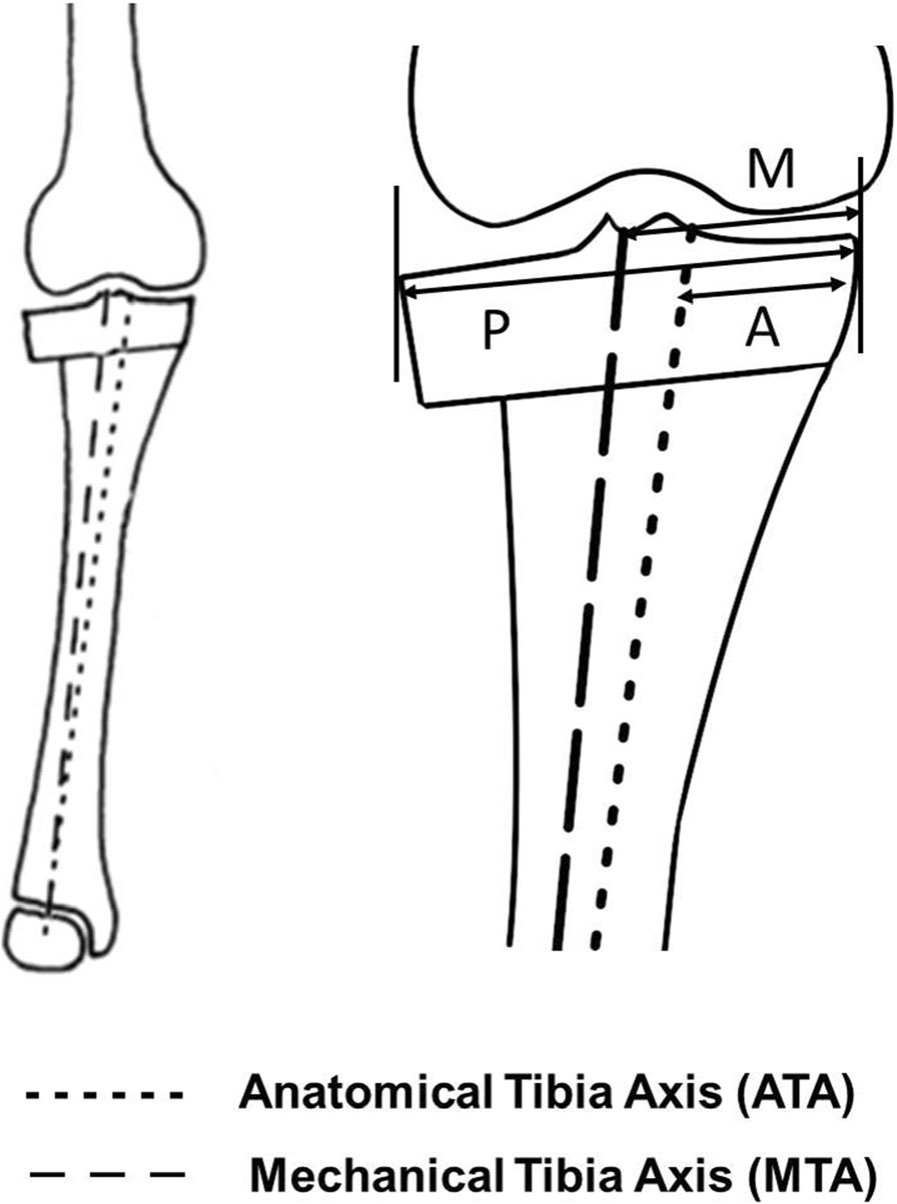
Definition of tibial axis deviation. The percentages of anatomical tibial axis deviation (%ATAD) and mechanical tibial axis deviation (%MTAD) are defined as the ratio of the distance from the medial edge of the proximal tibia to the passing points of the anatomical axis and mechanical axis on the tibial surface (A and M) and to the width of the proximal tibia (P), respectively. The percentage is calculated by multiplying this ratio by 100%

Radiographic assessment was independently performed by two orthopaedic surgeons. Anteroposterior radiographs of the knee were taken in the standing position preoperatively and 1 month postoperatively. Limb alignment was expressed as the femorotibial angle (FTA), measuring the lateral angle between the anatomical femoral and tibial axes [2]. The joint line convergence angle (JLCA) was measured as the angle formed between a line tangent to the distal femoral condyle and the proximal tibial plateau [20]. Full-length anteroposterior radiographs of the lower limb were taken in the standing position preoperatively and 1 month postoperatively. The mechanical medial proximal tibial angle (mMPTA) was measured as the medial angle formed between the tibial mechanical axis and the knee joint line of the tibia [21]. The percentage of mechanical axis deviation (%MAD) was defined as the ratio of the distance from the medial border of the proximal tibia to the mechanical axis of the lower limb to the width of the proximal tibia [22]. The percentage of anatomical tibial axis deviation (%ATAD) was defined as the ratio of the distance from the medial border of the proximal tibia to the passing point of the anatomical axis on the tibial surface to the width of the proximal tibia (Fig. 1). The percentage of mechanical tibial axis deviation (%MTAD) was defined as the ratio of the distance from the medial border of the proximal tibia to the passing point of the mechanical axis on the tibial surface to the width of the proximal tibia (Fig. 1). All radiographic images were collected with digital acquisition, and Fujifilm OP-A® software (Fujifilm, Co Ltd., Tokyo, Japan) was used for all measurements. The amounts of changes from preoperative to postoperative in the FTA, JLCA, mMPTA, %MAD, %ATAD, and %MTAD were defined as ΔFTA, ΔJLCA, ΔmMPTA, %ΔMAD, %ΔATAD, and %ΔMTAD, respectively. Comparisons of radiographic measurements between CWHTO and OWHTO were carried out to assess difference in each change of FTA for anatomical alignment, JLCA for soft tissue correction, %ATAD and %MTAD for shift of bony axes, and %MAD for location of the lower limb mechanical axis on the knee.

### Statistical analysis

Statistical analysis was carried out using BellCurve for Excel version 2.21 (Social Survey Research Information, Tokyo, Japan). The Mann-Whitney U test was used to compare the measurements between two different HTO procedures. The Wilcoxon signed-rank test was used to compare the measurements in preoperative planning between different HTO procedures in the same subjects. An adjusted *P* value < 0.05 was considered significant. A power calculation indicated that a sample size of 47 in each osteotomy procedure could detect differences with an effect size of 0.6, with 5% probability of a type I error and power of 80%. Thus, the required sample was determined to be 50 knees in each group. The intra-rater and inter-rater reliabilities of radiographic measurements were assessed by calculating intraclass correlation coefficients (ICCs).

## Results

### Clinical assessment

Mean surgical time was 152.3 ± 33.7 min for CWHTO and 113.2 ± 26.8 min for OWHTO (*P* <  0.05). Postoperative complications included 3 cases of deep venous thrombosis, 1 case of pulmonary embolism, 1 case of wound infection, and 1 case of peroneal nerve palsy in CWHTO, and 4 cases of deep venous thrombosis and 1 case of pulmonary embolism in OWHTO. The JOA score was significantly improved from preoperative 65.7 ± 8.5 to postoperative 89.2 ± 8.1 in CWHTO (*P* <  0.05), and from preoperative 71.4 ± 10.5 to postoperative 91.6 ± 6.9 in OWHTO (P <  0.05). There was no significant difference in the postoperative JOA score between CWHTO and OWHTO.

### Radiographic measurements

Pre- and postoperative measurements of FTA, %MAD, %ATAD, %MTAD, %MTAD - %ATAD, mMPTA, and JLCA are summarized in Table 2. There were significant differences in preoperative FTA, %MAD, and JLCA between CWHTO and OWHTO (*p* <  0.05). The mean FTA and JLCA decreased postoperatively, and the mean %MAD and mMPTA increased postoperatively in both CWHTO and OWHTO (*P* <  0.05). The mean postoperative %ATAD decreased in CWHTO (P <  0.05), which was significantly smaller than in OWHTO (P <  0.05). The mean postoperative %MTAD - %ATAD increased in CWHTO (*P* <  0.05), significantly greater than in OWHTO (P <  0.05).

**Table 2:** Radiographic measurements

		CWHTO	OWHTO
FTA (°)	Preop.	187.3 ± 8.7†	181.5 ± 5.5
	Postop.	167.8 ± 8.8*	167.8 ± 6.2*
%MAD	Preop.	−10.9 ± 32.3†	12.3 ± 25.1
	Postop.	72.3 ± 32.5*	75.6 ± 24.0*
%ATAD	Preop.	52.1 ± 14.4	50.5 ± 6.1
	Postop.	40.1 ± 11.8*†	49.7 ± 6.0
%MTAD	Preop.	49.8 ± 7.2	49.7 ± 5.6
	Postop.	51.2 ± 6.6	49.9 ± 6.4
%MTAD-%ATAD	Preop.	−2.4 ± 6.9	−0.8 ± 5.7
	Postop.	11.0 ± 7.6*†	0.2 ± 2.5
mMPTA (°)	Preop.	82.1 ± 9.9	83.8 ± 6.8
	Postop.	97.9 ± 8.1*	96.6 ± 8.6*
JLCA (°)	Preop.	6.0 ± 8.0†	3.6 ± 4.4
	Postop.	3.6 ± 5.4*	3.0 ± 3.0*

* P <  0.05 vs Pre-op † P <  0.05 vs CWHTO

FTA femorotibial angle, %MAD percentage of mechanical axis deviation, %ATAD percentage of anatomical mechanical axis deviation, %MTAD percentage of tibial mechanical axis deviation, mMPTA mechanical medial proximal tibial angle, JLCA joint line convergence angle, Preop. preoperative, Postop. postoperative

Differences between preoperative and postoperative radiographic measurements are summarized in Table 3. Magnitudes of ΔFTA, Δ%MAD, Δ%ATAD, Δ%MTAD, Δ(%MTAD - %ATAD), and ΔJLCA were significantly greater in CWHTO than in OWHTO (P <  0.05).

**Table 3:** Differences between pre- and postoperative radiographic measurements

	CWHTO	OWHTO	*P* value
ΔFTA (°)	−19.6 ± 10.6	−13.9 ± 7.1	< 0.001
Δ%MAD	83.2 ± 37.2	63.3 ± 26.2	< 0.001
Δ%ATAD	−12.0 ± 11.4	−0.8 ± 4.8	< 0.001
Δ%MTAD	1.4 ± 5.1	0.2 ± 4.8	0.05
Δ(%MTAD-%ATAD)	13.4 ± 9.5	1.0 ± 4.1	< 0.001
ΔmMPTA (°)	15.8 ± 7.8	12.8 ± 6.8	< 0.001
ΔJLCA (°)	−3.2 ± 3.8	−0.9 ± 3.1	< 0.001

FTA femorotibial angle, %MAD percentage of mechanical axis deviation, %ATAD percentage of anatomical mechanical axis deviation, %MTAD percentage of tibial mechanical axis deviation, mMPTA mechanical medial proximal tibial angle, JLCA joint line convergence angle

The ICCs for inter-and intra-rater reliabilities were all > 0.8, ranging from 0.88 to 0.97 for all radiological measurements, indicating good reliability.

### Effects of bony correction on mechanical axis shift and joint line inclination

To assess the effects of the bony correction angle on mechanical axis shift and joint line inclination, the ratios of Δ%MAD, ΔJLCA, or Δ(%MTAD - %ATAD) to ΔmMPTA were compared between CWHTO and OWHTO (Table 4). CWHTO resulted in a greater decrease of ΔJLCA/ΔmMPTA than OWHTO (*p* <  0.05), and a greater increase of Δ(%MTAD - %ATAD)/ΔmMPTA than OWHTO (*p* <  0.05). However, no significant difference was found in the Δ%MAD/ΔmMPTA between CWHTO and OWHTO. These results suggested a greater variation between the tibial anatomical and mechanical axes, and greater soft tissue correction in CWHTO compared to OWHTO, but the ratio of MAD change to the correction angle was comparable in both HTO procedures.

**Table 4:** Relationship between ΔMPTA and Δ%MA, ΔJLCA, or Δ(%MTA-%ATA)

	CWHTO	OWHTO	*P* value
Δ%MAD/ΔmMPTA	5.4 ± 4.3	4.9 ± 0.6	0.129
ΔJLCA/ΔmMPTA	−0.19 ± 0.28	− 0.05 ± 0.22	< 0.001
Δ(%MTA-%ATA)/ΔmMPTA	0.94 ± 0.83	0.06 ± 0.36	< 0.001

%MAD percentage of mechanical axis deviation, %ATAD percentage of anatomical mechanical axis deviation, %MTAD percentage of tibial mechanical axis deviation, mMPTA mechanical medial proximal tibial angle, JLCA joint line convergence angle

### Comparison of alignment changes between CWTHO and OWHTO in preoperative planning

**Fig. 2:**
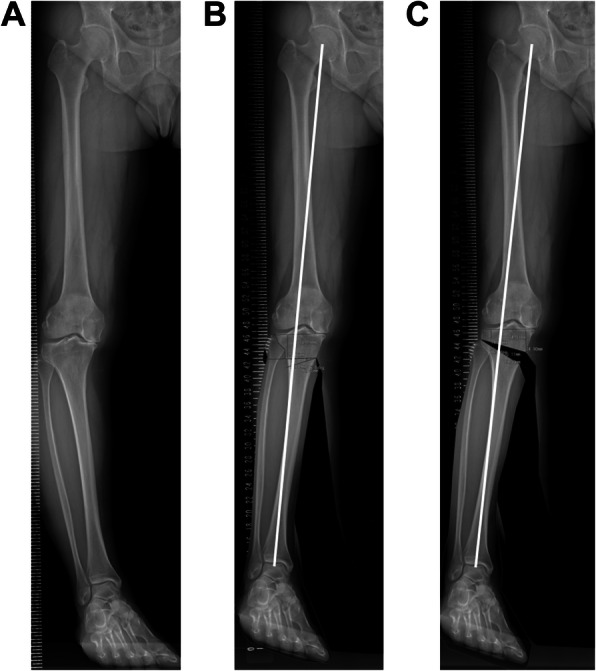
Preoperative planning for OWHTO or CWHTO with the same correction angle. The osteotomies are planned with a bony correction angle of 15° in a case with %MA of 0 (**a**), expecting %MA of 60 for CWHTO (**b**) and 68 for OWHTO (**c**)

To assess the difference in alignment change between CWTHO and OWHTO in preoperative planning, the cases that underwent CWHTO surgery were re-planned for CWHTO and OWHTO with the same correction angle according to the actual bony correction angle (ΔmMPTA) (Fig. 2). Changes of %MAD and mMPTA were significantly greater in OWHTO than in CWHTO (Table 5).

**Table 5:** Comparison of alignment changes between CWTHO and OWHTO in the preoperative planning by the same correction angle

	CWHTO	OWHTO	P value
Δ%MAD	61.6 ± 16.5	66.2 ± 17.9	< 0.001
ΔmMPTA	14.6 ± 3.8	15.4 ± 4.0	< 0.001

%MAD percentage of mechanical axis deviation, mMPTA mechanical medial proximal tibial angle

## Discussion

The most important finding of the present study was that CWHTO had a greater medial shift of the tibial axis and a lower valgus bony correction ratio than OWHTO. However, actual postoperative alignment was comparable between the two procedures due to greater valgus compensation of soft tissue in CWHTO.

**Fig. 3:**
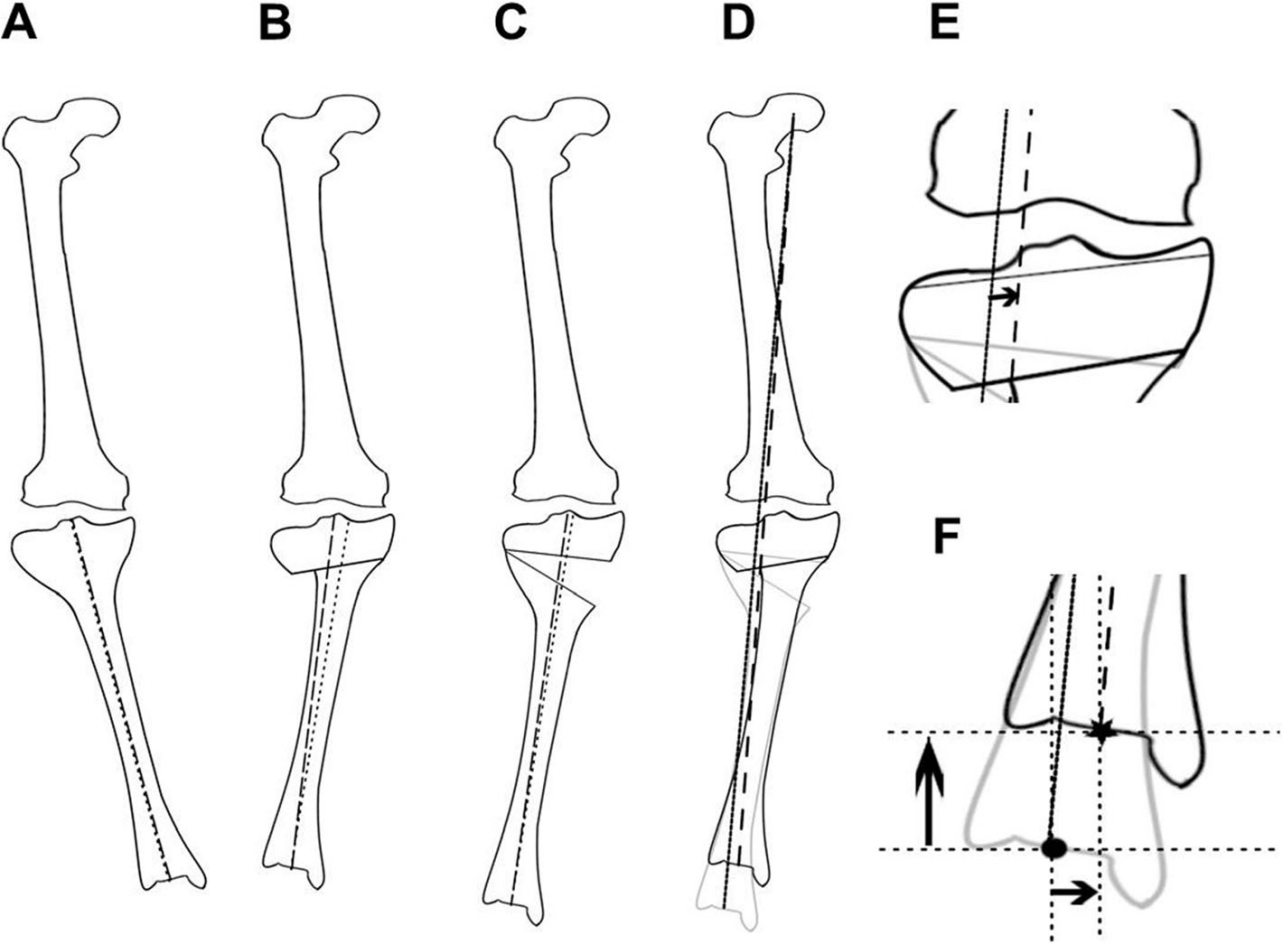
A schematic comparison between CWHTO and OWHTO in the position of the mechanical tibial axis including the centre of the ankle joint after osteotomies with the same bony correction angle. Illustrations show preoperative status (**a**), CWHTO (**b**), OWHTO (**c**), and an overlay of the two procedures (**d**). The mechanical axis passes more medially after CWHTO than after OWHTO (**e**). The centre of the ankle is positioned more medially and proximally after CWHTO (dot) than after OWHTO (asterisk) (**f**)

Lateral tibial condylar offset is created by HTO, and some transposition to the bony axis occurs. In general, CWHTO has greater lateral shift of the proximal tibia from the anatomical axis than OWHTO [10, 11], which often makes it difficult to perform revision total knee arthroplasty [23]. The present study confirmed the greater variation between postoperative tibial mechanical and anatomical axes in CWHTO, with almost no difference between them in OWHTO. Since the difference between these two axes on the tibial plateau increased by an average 1% of deviation for every 1° of correction angle in CWHTO, it may have potential for correction loss in cases with larger correction. Lateral tibial condylar offset also affects the amount of the correction angle in the different osteotomy procedures. Lateral tibial condylar offset after CWHTO resulted in medial shift of the tibial shaft including the ankle joint. In addition, leg length shortening also affects the medial shift of the ankle joint. In contrast, the medial shift of the tibial shaft was relatively small, and leg length was extended after OWHTO. Thus, the mechanical axis of the lower limb on the tibial surface would pass more laterally in OWHTO than in CWHTO, when the osteotomy is performed with the same bony correction angle (Fig. 3). If the same target alignment is preoperatively planned in both OWHTO and CWHTO, CWHTO requires a greater correction angle than OWHTO.

When preoperative planning for CWHO and OWHTO was carried out by the same correction angle, mMPTA was significantly increased in OWHTO than in CWHTO (Table 5). Consequently, %MAD was expected to increase significantly in OWHTO, suggesting overcorrection. However, the present study demonstrated that the actual postoperative lower limb alignment in OWHTO was comparable with that in CWHTO. Dugdale et al. have shown that total varus angulation of the OA knee was composed of three potential components: femorotibial geometric alignment, narrowing or loss of the osteocartilaginous complex, and separation of the lateral joint due to slack ligamentous and soft tissues [24]. Lower limb alignment after HTO is affected by soft tissue balance, as well as the bony correction angle [25, 26]. Unexpected valgus overcorrection may be due to large preoperative JLCA in both OWHTO [20] and CWHTO [27]. A larger correction angle also affects overcorrection [25]. In the present series, CWHTO showed a greater ratio of JLCA change to the bony correction angle than OWHTO. Although the preoperative planning of CWHTO is likely to indicate undercorrection compared to that of OWHTO with the same correction angle, in fact, larger postoperative change of the JLCA compensates the valgus angle in CWHTO, and total alignment is equivalent in both osteotomy procedures.

The differences between the two techniques are still controversial [8, 9, 28, 29], and there is still no precise indication for either technique. Ferner et al. introduced a unique algorithm for choosing between the HTO procedures, OWHTO or CWHTO, based on torsional deformity, patellar height, and length discrepancy [30]. In the clinical setting, the extent of the correction angle should be one of the most important factors for choosing either OWHTO or CWHTO. The correction angle is limited to 15° or less in OWHTO [7], whereas a larger correction is possible in CWHTO. Either technique can be selected in the borderline cases with a correction of around 15°.

Furthermore, there was some controversy about whether OWHTO or CWHTO could achieve the planned correction more accurately. Radiological outcomes in a randomized trial comparing CWHTO and OWHTO did not show a difference in postoperative coronal alignment between the two HTO procedures [9]. However, the other prospective randomized trial comparing two different HTO techniques demonstrated that CWHTO achieves a more accurate planned correction than OWHTO [31]. In contrast, several studies showed that OWHTO provided higher accuracy of correction than CWHTO [32, 33]. There may be several reasons for the controversy about accuracy between the two HTOs, including differences in osteotomy technique, fixation device, and correction angle [11, 34–36]. The present results showed that there was no significant difference in postoperative lower limb alignment between CWHTO and OWHTO, although there was a discrepancy in the preoperative planning with the same correction angle. This result may be due to a combination of several factors including correction angle, extent of tibial axis shift, and additional correction by soft tissue.

This study has several limitations. The follow-up time for radiographic assessment of 1 month was short. The current series assessed the early postoperative change of knee alignment. The indication for either HTO technique was determined preoperatively according to the correction angle. This may affect the amount of change in JLCA.

## Conclusion

The ratio of mechanical axis shift to the correction angle differed in preoperative planning, but actual postoperative alignment was comparable between OWHTO and CWHTO. This study supported the part of the initial hypotheses that CWHTO shows greater medial shift of the tibial axis, but did not support the other part regarding less mechanical axis change than in OWHTO with the same bony correction angle. The discrepancy occurred due to greater soft tissue correction in CWHTO compared to OWHTO. Therefore, planning of the correction angle for target alignment needs to take into account the effect of soft tissue correction as well as bony correction.

## Data Availability

The datasets used and/or analysed during current study are available from the corresponding author on reasonable request.

## Abbreviations

FTA: femorotibial angle
%MAD: percentage of mechanical axis deviation
%ATAD: percentage of anatomical mechanical axis deviation
%MTAD: percentage of tibial mechanical axis deviation
mMPTA: mechanical medial proximal tibial angle
JLCA: joint line convergence angle
